# Alpha-Mangostin: A Review of Current Research on Its Potential as a Novel Antimicrobial and Anti-Biofilm Agent

**DOI:** 10.3390/ijms26115281

**Published:** 2025-05-30

**Authors:** Hanna Górecka, Mateusz Guźniczak, Igor Buzalewicz, Agnieszka Ulatowska-Jarża, Kamila Korzekwa, Aleksandra Kaczorowska

**Affiliations:** 1 Department of Biomedical Engineering, Faculty of Fundamental Problems of Technology, Wrocław University of Science and Technology, 27, Wybrzeże S. Wyspiańskiego St., 50-370 Wrocław, Poland; 261915@student.pwr.edu.pl (H.G.); mateusz.guzniczak@pwr.edu.pl (M.G.); igor.buzalewicz@pwr.edu.pl (I.B.); agnieszka.ulatowska-jarza@pwr.edu.pl (A.U.-J.); 2 Department of Microbiology, Faculty of Biological Sciences, University of Wrocław, 63, Przybyszewskiego St., 51-148 Wrocław, Poland; kamila.korzekwa@uwr.edu.pl

**Keywords:** *Garcinia mangostana*, α-mangostin, biofilm, antibacterial effect, phytotherapeutics, phytochemicals, xanthone

## Abstract

Alpha-mangostin (α-MG) is a prenylated xanthone extracted from the pericarp of the mangosteen tree (*Garcinia mangostana*) fruit. The compound exhibits a broad range of therapeutic properties, such as anti-inflammatory, antioxidative, and antimicrobial activity. This review highlights new findings in antibacterial studies involving α-MG, demonstrates its potent activity against Gram-positive bacteria, including *Staphylococcus* and *Enterococcus* genera, and describes the antibacterial mechanisms involved. Most cited literature comes from 2020 to 2025, highlighting the topic’s relevance despite limited new publications in this period. The primary antibacterial mechanism of α-MG consists of the disruption of the bacterial membrane and increased bacterial wall permeability, leading to drug accumulation and cell lysis. Other mechanisms include genomic interference and enzyme activity inhibition, which impair metabolic pathways. α-MG can also disrupt biofilm formation, facilitate its removal, and prevent its maturation. Furthermore, α-MG presents strong synergistic action with common antibiotics and other phytochemicals, even against drug-resistant strains, facilitating infection treatment and allowing for reduced drug dosage. The main challenge in developing α-MG-based drugs is their low aqueous solubility; therefore, nanoformulations have been explored to improve its bioavailability and antibacterial stability. Extended research in this direction may enable the development of effective antibacterial and anti-biofilm therapies based on α-MG.

## 1. Introduction

Herbal medicine has been used by humans since antiquity and is still popular worldwide. In recent years, organic natural products have gained interest in the context of potential use in various treatments. In the years 1981–2014, around 51% of newly approved small-molecule drugs were of natural origins [1]. There is a strong belief that natural substances, many of which remain unexplored, hold significant potential for anticancer, antimicrobial, anti-inflammatory, and other therapeutic properties. The plant kingdom offers a wide variety of bioactive compounds (phytochemicals) that can be used for therapeutic purposes. Although extracting these compounds can be challenging, it has been proven that simply consuming fruits and vegetables can lower the chances of cancer [2]. The search for a treatment option for both cancer and infections leads scientists to examine the exact applications of known phytochemicals.

One of the compounds that have gained researchers’ attention is α-mangostin (α-MG), a phytochemical derived from a fruit native to Southeast Asia. The fruit of the mangosteen tree (*Garcinia mangostana*) is edible and considered a delicacy. The fruit has dark purple skin and contains soft white pulp, which has a sweet and acidic taste [3]. The fruit’s pericarp has been used in traditional medicine in Thailand, India, Sri Lanka, and Myanmar as a cure for different maladies, varying from diarrhea to wounds and skin infections [4]. The anti-inflammatory properties of mangostin have proven useful in these applications. Today, α-MG is widely studied in the context of antimicrobial and drug delivery purposes.

Mangosteen fruit contains numerous phytochemicals, of which α-MG is the most notable. α-MG is a xanthone, a secondary metabolite found in natural products derived from plants. Xanthones have been reported to be highly viable active ingredients with antioxidative, anti-inflammatory, antiallergic, and antimicrobial properties. Other compounds from the same group (β-mangostin, γ-mangostin, gartanin, garcinone E, and 8-deoxygartanin), in varying concentrations, can be found in the pericarp of the fruit. Studies suggest that these compounds present similar biological activities to α-MG but are often weaker in comparison [5,6].

To extract α-MG, the mangosteen fruit peel is used. Many methods of extraction have been tested, including classical techniques (Soxhlet extraction, maceration, decoction), modern assisted methods (microwave-assisted, ultrasonic-assisted, supercritical fluid and ${\mathrm{CO}}_{2}$ extraction, biphasic micellar systems, eutectic solvents), and solvent-based extraction using hexane, chloroform, ethanol (various concentrations), and methanol [6]. The choice of extraction method is crucial for optimizing yield and purity. The resulting substance is a yellow, bitter powder with low water solubility [7]. It is, however, soluble in organic solvents such as ethanol [6,7].

The field of phytochemical treatment research is rapidly evolving, with new studies published every year. However, few papers focus on the antibacterial activity of α-MG. A search for related articles in the last five years (from 2020 to 2025) has shown that only 51 papers are available in Google Scholar, which were published during this period, containing “α-mangostin” in their title and either “bacteria” or “antibacterial” in their keywords. Even fewer articles were found that included “biofilm” in their keywords. Such criteria for the search were intentionally selected to ensure relevance, because broader search terms (e.g., searches not limited to titles containing “α-mangostin”) resulted in unrelated publications on other compounds or different applications of α-MG. The PubMed database has proven even more limited in relevant publications. Because of that, the search criteria were adjusted to be less restrictive, and one search query did not require the publication title to contain “α-mangostin” in its title. Even then, only 29 articles were identified. The search results are summarized in Table 1. The performed research on related publications proved that the topic remains unexplored and requires more attention.

This review aims to summarize recent advances in the study of α-MG, highlighting its therapeutic potential in antimicrobial drug development. The focus of this article is describing the antibacterial mechanisms of action of α-MG as well as its synergistic and anti-biofilm applications.

## 2. Antibacterial Applications of α-Mangostin

The emerging crisis of drug-resistant microbes brings scientists’ attention to research on new methods of treating infections. Antimicrobial resistance (AMR) causes previously used antimicrobials and antibiotics to be insufficient. Higher doses of known drugs might be administered to patients, risking reaching a high toxicity level. This is a serious threat to the population worldwide, as 700,000 people are estimated to die because of drug-resistant microbes every year [9]. It is predicted that unless serious preventive actions are taken, the death toll will increase, making AMR-related infections the main cause of mortality in the world [10].

AMR is most often mentioned in the context of bacteria because of multidrug-resistant bacteria strains such as *Staphylococcus aureus*, *Escherichia coli*, and *Klebsiella pneumonia* being widespread in the clinical environment. This phenomenon is caused mainly by human activity. Although AMR can occur naturally, unsafe practices related to antimicrobial drug use are greatly enhancing this process. Many sectors of human activity influence this, ranging from the treatment of infections with antibiotics in the health sector to feeding them to animals as part of livestock agriculture [11].

To deal with the AMR crisis, new antimicrobial solutions are being researched, with the focus shifting toward novel technologies involving nanoparticles, targeted drugs, and physicochemical methods. Research into natural compounds is also producing promising results. Utilizing the natural defense mechanisms of plants against microorganisms provides a means to overcome the challenge of AMR. Many phytochemicals have been shown to have antimicrobial activity [12]. In this section, we provide an overview of the research on the antibacterial properties of α-MG as a promising plant-based approach. The following sections will provide a detailed explanation of the antibacterial mechanisms of α-MG’s actions. Then, the effects on different bacterial strains will be compared and described.

Compounds of plant origin exhibit antimicrobial activity. The growing problem of antibiotic resistance has drawn attention to the use of natural agents, such as α-MG, as antimicrobial agents.

### 2.1. Chemical Structure of α-Mangostin

**Figure 1:**
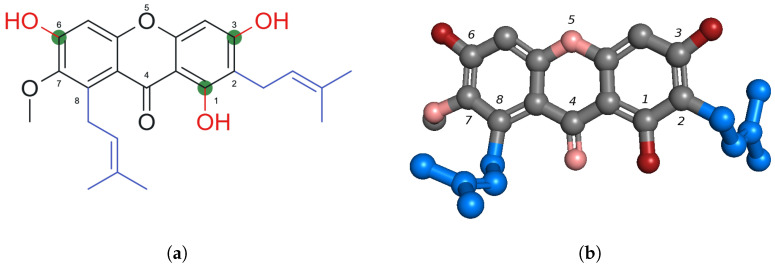
Comparison of 2D and 3D structures of α-mangostin (α-MG) with atom numbering according to IUPAC convention: (**a**) 2D chemical structure of α-MG with key functional groups: hydroxyl groups (red), hydrogen bonding sites (green), hydrophobic prenyl chains (blue). Image prepared by the authors using MarvinSketch (ChemAxon) based on [13]. (**b**) A 3D ball-and-stick representation of α-MG, colored by atom type (carbon (gray), oxygen (pink)) and key functional groups (hydroxyl groups (red), hydrophobic prenyl chains (blue)). The structure was visualized in PyMOL.

The antibacterial activity of α-MG is largely attributed to its chemical structure. α-MG’s molecular formula is $C_{24}H_{26}O_{6}$ and its structure is shown in Figure 1 [6]. The compound is a prenylated xanthone, structurally classified as a 1,3,6,7-tetraoxygenated xanthone bearing two isoprenyl ($C_{5}$) side chains at the C-2 and C-8 positions. This unique substitution pattern has been associated with high antibacterial and antimycobacterial activity [13].

The combination of hydrophilic hydroxy groups and lipophilic isoprenyl chains contributes to α-MG’s strong affinity for lipid bilayers, enabling membrane interaction and penetration [13,14]. Its strong affinity for hydrophobic alkyl chains in the bacterial membrane has also been proposed as a major factor enabling this activity [15]. The C-3 and C-6 hydroxyl groups have been shown to be the main interaction sites between α-MG and the bacterial wall [16]. In addition, structural modifications at the C-3 position, such as allyl substitution, have been shown to enhance its interaction with bacterial resistance proteins like penicillin-binding protein 2a (PBP2a) [17].

The efficacy of xanthone action against bacteria is closely tied to its chemical structure. Additionally, it has been noted that the strong activity against Gram-positive bacterial strains correlates with the presence of hydroxyl groups at positions C6 and C3, as well as a prenyl chain at position C2.

### 2.2. Mechanisms of Antibacterial Action

Many antibacterial mechanisms have been recorded for α-MG. The range of these mechanisms allows for α-MG to be used for bactericidal applications in many fields, such as dentistry, dermatology, and biofilm prevention.

**Figure 2:**
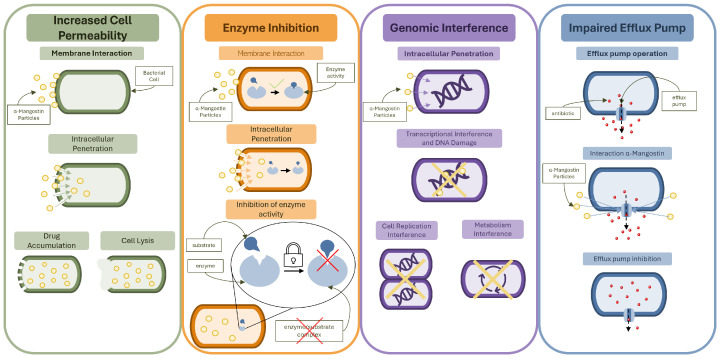
Summary of α-MG antibacterial mechanisms against Gram-positive bacteria.

The multi-target mode of antibacterial action allows for comprehensive activity, leading to strong effects against Gram-positive bacteria. The mechanisms are further explained in detail in the following sections and summarized in Figure 2. The mechanisms presented in Figure 2 are not only individually bactericidal, but complement each other and influence the overall physiology of bacterial cells. This is also important in α-MG’s synergistic effect when combined with antibiotics and other phytochemicals.

Compounds of natural origin, including those derived from plants, often present more than one mode of biological activity. Because of that, they can influence multiple targets simultaneously and be used in various fields.

#### 2.2.1. Membrane Disruption and Cell Wall Permeability

It has been shown that α-MG can penetrate the bacterial wall, disrupting its structure and thus leading to cell death. This is most effective against the Gram-positive bacteria due to their thinner membrane in comparison to Gram-negative bacteria. In a study by Jun-Jie Koh et al. [15], the impact of α-MG on methicillin-resistant *Staphylococcus aureus* (MRSA), *Bacillus cereus*, and *Enterococcus faecalis* has been studied. All of these were Gram-positive bacteria. It was shown that the minimum inhibitory concentration (MIC) of 0.78–1.56 μg/mL was effective against different strains of methicillin-sensitive *S. aureus* (MSSA), MRSA, and *B. cereus*. The MIC for α-MG was much lower than for other xanthones isolated from *G. mangostana*, for which the MIC ranged from 3.125 to 12.5 μg/mL, some of which were entirely ineffective. The bactericidal action of α-MG was rapid, with even 5 min of interaction causing a significant (3 log CFU/m) reduction in viable cell count. This aligns with the mechanism of bacterial membrane disruption. Membrane depolarization, resulting in ion homeostasis disruption and membrane damage, in the presence of at least the MIC of α-MG has been shown in MRSA [15].

The ability of α-MG to penetrate the bacterial wall has also been proven in a 2022 article by Felix et al. [18]. In this study, the effect, studied by analyzing the relative fluorescence, proves that α-MG penetrates the cell wall within 30 min of interaction, as demonstrated by increased SYTOX Green dye uptake in bacterial cells following treatment. Increased membrane permeability for MRSA strains has been shown for a MIC of 2 μg/mL by Felix et al. [18] and a higher concentration of 7.8 μg/mL by Meah et al. [19].

Increased permeability of the cell membrane alters the osmotic balance of the cell, causing external agents, such as water, ions, and drug particles, to penetrate the inside of the cell. This can result in leakage of intracellular matter because of increased internal pressure [20]. However, another important effect is that external molecules, such as antibiotics, can enter through the permeabilized membrane into the inside of the cell [15]. This influx bypasses the selective permeability barrier that normally protects the bacterial cell from exogenous threats. As a result, bacterial homeostasis is disrupted, ultimately leading to irreversible cellular damage and death.

A key factor responsible for this permeability increase is the strong hydrophobic interaction between α-MG and the lipid bilayer. The compound’s high affinity for the membrane’s alkyl chains perturbs lipid packing, leading to structural destabilization and leakage of cellular contents [15]. The membrane disruption can be proven by analyzing the absorbance at 260 nm, using a UV-Vis spectrophotometer, as shown by Meah et al. [19]. This method relies on UV absorption of intracellular matter. This allows for the quantification of leaked material by analyzing the increase in absorption [21]. Another method for determining the membrane damage is imaging, as shown by Koh et al. [15], who used scanning electron microscopy (SEM) images of *S. aureus* after 30 min incubation with α-MG to show induced cell lysis and membrane disruption. This method was also used by Sivaranjani et al. in the study of *Staphylococcus epidermidis* [22]. Field-emission SEM has also been used [16]. Other studies on membrane disruption in bacteria used such imaging methods as fluorescence microscopy, where the selective staining of alive and dead bacteria allowed for visual differentiation of the cells [23], or cryo-electron tomography combined with high-speed atomic force microscopy, which allows for a time-dependent structural analysis [24].

The main difference in the activity of α-MG against Gram-positive and Gram-negative bacteria can be related to the structure of the outer membrane of these bacteria and the presence of an additional membrane in Gram-negative bacteria. This provides a lower permeability for various active compounds.

#### 2.2.2. Inhibition of Bacterial Enzymes and Metabolic Pathways

In addition to its membrane-targeting effects, α-MG can inhibit enzyme activity. This can disrupt key intracellular metabolic processes. In a 2011 article, Nguyen and Marquiz [25] demonstrated that α-MG inhibits glycolysis in *Streptococcus mutans*, a Gram-positive, cariogenic oral bacterium. Since cariogenicity is linked to glycolysis and acid production, they examined how α-MG affects this pathway by targeting enzymes such as lactate dehydrogenase, aldolase, and glyceraldehyde-3-phosphate dehydrogenase at concentrations as low as 14 μmol/L. Furthermore, the glycolysis inhibition appeared to be irreversible, as washing the cells did not bring back the earlier rates of glycolytic capacity. This suggests a stable intracellular interaction.

The compound also blocked the activity of the membrane-bound $F_{1}F_{0}$-ATPase, an enzyme complex essential for adenosine triphosphate (ATP) synthesis, in both previously permeabilized and intact cells, which confirmed that α-MG effectively reaches cytoplasmic targets. Because of ATP’s fundamental role in cellular energy production, its inhibition can lead to cell death and is suggested to be the main bactericidal mechanism by the study’s authors. Interestingly, the study also showed that $F_{0}$ is the enzyme component sensitive to α-MG, which is consistent with the membrane-targeted actions of α-MG since $F_{0}$ is embedded in the cell membrane while $F_{1}$ extends into the cytoplasm [25,26]. The inhibitory effect on ATP synthesis was further supported by Park et al. [16], who demonstrated that α-MG significantly downregulated the expression of membrane-associated proteins involved in proton gradient maintenance and energy metabolism. The suppression of these proteins likely contributes to the collapse of ATP production and membrane homeostasis. Furthermore, α-MG inhibits the phosphotransferase system (PTS), involved in sugar (fructose, sucrose, lactose, and mannose) uptake, with 50% inhibition at 42 μmol/L (17.24 μg/mL) [25].

In addition to the direct inhibition of metabolic enzymes, α-MG also modulates the transcriptional activity of genes responsible for biosynthesis and replication. In a 2019 study, Sivaranjani et al. [27] used omic technologies to study α-MG interaction with *Staphylococcus epidermidis*, a Gram-positive bacterium associated with both hospital- and community-acquired infections. The study confirmed that the α-MG mechanism of killing bacteria is related to compromising membrane integrity by downregulating genes and proteins associated with the cytoplasmic membrane (YidC2, SecA, FtsY, and MscL). It was also observed that α-MG influenced the cell’s basic genetic processes, such as DNA replication and repair, by inhibiting DNA metabolism genes. These genes included DNA polymerases (*polA*, *polC*, and *dnaE*), which are essential for chromosomal DNA replication, as well as *uvrA* and *mfd* genes, responsible for DNA repair mechanisms. A gene linked to the transportation of teichoic acids through the membrane (*tagG*) was also downregulated. Because of teichoic acids’ importance in the Gram-positive bacteria’s cell wall, this influences the structural integrity and surface properties, which may increase surface charge density and enhance α-MG binding.

Another finding presented by Sivaranjani et al. [27] is that the downregulation of the *secA1* and *secA2* genes, related to toxin secretion, contributes to bypassing and weakening the cell’s defense system, including efflux-related mechanisms. As reported by Felix et al. [18], genes directly linked with efflux pumps can also be affected in *S. aureus* MRSA strains. In their study, Felix et al. showed that among the genes downregulated by α-MG were *norA* and *norB*, which regulate efflux pumps linked to fluoroquinolones, such as ciprofloxacin and norfloxacin. Another study by Ge et al. [17] reported the downregulation of *norA* and *mepA*, both of which are efflux pump-related. Regulation of the *norA* gene might be especially important in the context of multidrug-resistant bacteria, such as MRSA, since one of the resistance mechanisms is upregulating this gene to increase drug excretion [28].

Another study showing α-MG’s DNA interference was conducted by Park et al. [16], who observed broad transcriptional repression of genes related to membrane integrity and metabolic regulation. Notably, α-MG reduced the expression of membrane-associated protein genes involved in homeostasis and energy-related processes, supporting its multi-targeted antibacterial mode of action at the transcriptional level.

#### 2.2.3. Activity Against Gram-Negative Bacteria

α-MG’s action against Gram-negative bacteria is very weak. This can be explained by α-MG’s antibacterial action described in three stages. In the first stage, it disrupts the cytoplasmic membrane, increasing its permeability. Then, the synthesis of enzymes responsible for cell wall initiation is inhibited, and lastly, damage to the peptidoglycan structure weakens the cell wall. This causes the cell wall’s depolarization and intracellular contents’ leakage [15].

Research shows that the activity of α-MG’s against Gram-negative bacteria is significantly weaker compared to Gram-positive bacteria. The main reason is the difference in the bacterial wall structure of Gram-negative and Gram-positive strains. Gram-positive bacteria have a thick, multilayered cell wall primarily composed of murein (peptidoglycan), which can constitute up to 70% of the dry mass. The cell wall of Gram-positive bacteria also contains teichoic and teichuronic acids, which extend above the murein layer, forming a thin polysaccharide coating and further reinforcing the cell wall. However, Gram-negative bacteria possess a thin cell wall consisting of a single layer of murein, which constitutes only 10% of the dry mass. The cell wall is surrounded by an outer membrane that contains lipopolysaccharide (LPS), phospholipids, and proteins. This outer membrane and the hydrophobic lipopolysaccharides, which it contains, effectively protect the Gram-negative bacteria, forming a selectively permeable barrier for hydrophilic substances [29]. Furthermore, the membrane contains porin proteins that selectively facilitate the penetration of small, hydrophilic molecules, additionally hindering the penetration of larger or hydrophobic compounds. Another problem is the strong negative surface charge of the Gram-negative bacteria, which repels the α-MG molecules.

To increase α-MG’s activity against Gram-negative bacteria, including the penetration of the outer membrane, conjugation with cationic compounds or nanoparticles can be used [30,31]. Another method is synergistic therapy, where α-MG can be used in combination with antibiotics to improve or restore their effectiveness against multidrug-resistant bacterial strains. Even then, however, antibacterial action against the Gram-negative bacteria is weaker and requires prolonged therapy to observe the expected effects [32,33].

#### 2.2.4. MIC Comparison

Varying methods of extraction and treatment decisions can influence the results of antibacterial action. The MIC values presented in cited articles are presented in Table 2. Most studies show that the MICs of α-MG for Gram-positive bacteria are very low, regardless of the method used to measure the MIC, which confirms its strong antibacterial properties. In contrast, a significant difference is observed with Gram-negative bacteria, where the MIC is substantially higher or impossible to obtain [16,17,34]. This implies that α-MG exhibits no significant antibacterial effect against Gram-negative bacteria.

The primary reason for this disparity lies in the structural differences in the cell walls between Gram-negative and Gram-positive bacteria. Gram-positive bacteria lack an outer membrane, while Gram-negative bacteria have a thin peptidoglycan layer that is encased by an outer membrane rich in lipopolysaccharides. Since bacterial cell walls are the main target for α-MG, this difference in structure inhibits its ability to penetrate the membrane and effectively kill the bacteria.

Although the MIC for α-MG is possible to be established, as demonstrated previously, the situation is more complicated when it comes to the use of plant extract from *Garcinia mangostana*. Such extracts are not standardized and may contain varying concentrations of α -MG as well as other xanthones and other compounds that can affect the experimental results. Due to the complex composition of plant-derived products, it is difficult to standardize their extraction processes to ensure the potency, efficacy, and stable concentration of all compounds [35]. Different extraction processes may result in differences in chemical composition, which influence the extract’s bioactivity [36]. Nevertheless, efforts to unify the mangosteen extract are being conducted. Methods based on chemometrics and Fourier transform infrared spectroscopy or near-infrared spectroscopy have been proposed as a solution for quality assurance of such extracts [37,38].

Consequently, while some studies provide information about α-MG MIC when using *Garcinia mangostana* extract, such results should be critically evaluated. Even when α-MG’s concentration in the extract is known, the measurement does not account for other compounds’ influence on the antibacterial activity. MIC values of the *Garcinia mangostana* extract cannot be effectively evaluated either because of the lack of standardization. Although numerous articles report these MIC values [39,40,41,42,43], they can be more useful for comparison of the extract’s effects on different bacterial species rather than providing quantifiable or reliable MIC values.

Cases of α-MG use described in the literature show varying results in the MIC values. α-MG requires higher MIC doses when it is used to treat infections caused by Gram-negative bacteria.

**Table 2:** MIC values of α-MG from the literature against various bacterial strains.

Study	Bacterial Species and Strains	MIC (μg/mL)	Method	Solvent
Phitaktim et al. (2016) [14]	*S. aureus* ATCC 29213(+)	4	Broth microdilution	DMSO ^2^
	*ORSS strains* ^1^(+)	8		
Koh et al. (2013) [15]	*S. aureus* DM 21455(+)	1.56	Broth microdilution	DMF ^3^
	*S. aureus* DM 09808R(+)	1.56		
	*B. cereus* ATCC 11778(+)	1.56		
	MRSA DB 57964/04(+)	1.56		
	*S. aureus* ATCC 29213(+)	0.78		
Park et al. (2023) [16]	*S. aureus* ATCC 29213(+)	4	Agar dilution	DMSO ^2^
		2	Broth microdilution	
	*S. carprae* KCTC 3583(+)	2	Agar/broth microdilution	
	*S. epidermidis* ATCC 12228(+)	1	Agar/Broth microdilution	
	*S. felis* ATCC 49168(+)	2	Agar dilution	
		1	Broth microdilution	
	*S. intermedius* KCTC 3344(+)	2	Agar dilution	
		1	Broth microdilution	
	*S. pseudintermedius* ATCC 49051(+)	2	Agar dilution	
		1	Broth microdilution	
	*S. saprophyticus* KCTC 3345(+)	2	Agar dilution	
		1	Broth microdilution	
	*S. schleiferi* ATCC 43808(+)	1	Agar/Broth microdilution	
	*E. coli* ATCC 25922(−)	>64	Agar/Broth microdilution	
	*P. aeruginosa* ATCC 27853(−)	>64	Agar/Broth microdilution	
Ge et al. (2024) [17]	*S. aureus* ATCC 29213(+)	0.5–2	Broth microdilution	Not specified
	MRSA2(+)	0.5–2		
	*E. coli* ATCC25922(−)	>256		
	*E. coli* CRE-1(−)	>256		
Felix et al. (2022) [18]	*S. aureus* MRSA MW2(+)	2	Microdilution	DMSO ^2^
Meah et al. (2020) [19]	MRSA DMST 20654(+)	15.6	Microdilution	DMSO ^2^
	MRSA-142(+)	31.25		
	MRSA-2468(+)	31.25		
	MRSA-1096(+)	7.81		
Sivaranjani et al. (2017, 2019) [27,44]	*S. epidermidis* RP62A(+)	1.25	Microdilution	Not specified
Sakagami et al. (2005) [32]	VRE strains ^4^(+)	3.13–6.25	Agar dilution	DMSO ^2^
	VSE strains ^5^(+)	3.13–6.25		
	MRSA strains ^6^(+)	6.25–12.5		
	MSSA strains ^7^(+)	6.25		
Tangsuksan et al. (2022) [34]	*S. mutans* ATCC 25175(+)	117	Microdilution	Soluble film
	*P. gingivalis* ATCC 33277(−)	117		

(+)/(−) Gram-positive/negative bacteria. ^1^ *S. saprophyticus* DMST 27055, DMST 27058, DMST 4236, DMST 4672, DMST 5057, DMST 8034; ^2^ dimethyl sulfoxide; ^3^ N,N-dimethylformamide; ^4^ vancomycin-resistant *Enterococci* strains: *E. faecalis* ATCC 51299, ATCC 51575; *E. faecium* ATCC 51559, KIHC-237; *E. gallinarum* KIHC-241; ^5^ vancomycin-sensitive *Enterococci* strains: *E. faecalis* IFO 12965, ATCC 8459; *E. faecium* IFO 3535; ^6^ strains not specified; ^7^ methicillin-sensitive *S. aureus* strains: IFO 13276, IFO 12732, IFO 3080.

### 2.3. Anti-Biofilm Activity

Biofilms are complex communities of microorganisms encased in an extracellular polymeric matrix. Multi-species biofilms represent the most common natural ecosystems. The microorganisms within the biofilm exhibit different physiological and metabolic properties compared to planktonic organisms of the same species. [18,45,46,47]

Anti-biofilm solutions are sought after because of the infection rates of biofilms. Biofilms pose a threat to public health as they are one of the main causes of infections, with an estimated 65 to 80% of all infections being caused by biofilms [48]. They are present in healthcare settings by forming on surfaces in hospitals, medical equipment, and even surgical implants. Other industries influenced by biofilms include the food industry and aquaculture [47]. The concern is increased by the fact that biofilms are the most resilient forms of microorganisms. The bacteria in biofilms are 1000 times more resistant to antibiotics [48] and can become resistant to UV, dehydration, salinity, and other bactericidal environmental factors [47].

α-MG has demonstrated promising anti-biofilm activity through multiple complementary mechanisms. These included inhibiting biofilm growth and penetration through the extracellular matrix to reach the microorganisms and cause their death or damage [2,6,27].

It is currently known that bacteria can form an organized structure called biofilms. The highly structured nature of biofilms limits the penetration of antibiotics. This is why alternative methods of treatment are being researched to fight biofilms. These include substances based on natural compounds such as α-MG.

#### 2.3.1. Mechanisms of Anti-Biofilm Action

The initial adhesion of bacterial cells to a surface is a critical prerequisite for biofilm development, as it enables the establishment of microcolonies and the production of the extracellular matrix that stabilizes the biofilm as it matures. α-MG possesses the ability to interfere with the adhesion process during the early stages of biofilm formation as shown by Nguyen et al. [49], who studied the biofilm formation of *S. mutans* on saliva-coated apatitic surfaces. As a result of biofilm’s short-term treatment with α-MG at a concentration of 150 μmol/L, the inhibition of biofilm growth was observed. Furthermore, α-MG treatment affected the structural integrity of the biofilm, leading to more sparsely distributed microcolonies and areas completely devoid of colonies. The stability of the biofilm was compromised, as it proved to be easier to remove from the surface by shear force than the control sample. The inhibited adhesion to the surface was accompanied by lessened cell–matrix cross-linking forces and viscoelasticity, ultimately weakening the biofilm structure. This was a result of α-MG’s inhibition of the synthesis of extracellular polysaccharides, which were the crucial component of the biofilm’s matrix.

In addition, Sivaranjani et al. [22] demonstrated similar effects of α-MG on early-stage adhesion. Their findings revealed that treatment of *Acinetobacter baumannii* biofilms with α-MG at a concentration of 2 μg/mL inhibited biofilm growth during the initial stages by interfering with both adhesion and maturation processes. This could result from α-MG’s interference with the formation of cell-to-cell and cell-to-surface interactions and downregulating the *bfmR* and *csuA/B* genes responsible for initial biofilm surface adherence. Notably, no bactericidal action against the bacteria within the biofilm was observed. Moreover, α-MG reduced cell surface hydrophobicity by inhibiting the synthesis of the Bap protein, critical for biofilm development. The presence of Bap protein strongly influences the biofilm formation on various surfaces, most likely by facilitating intercellular and cell–surface binding [50].

The extracellular matrix stabilizes biofilms by enabling adhesion, cohesion, and protection. Its disruption compromises biofilm integrity, enhancing susceptibility to removal and external stressors such as antimicrobial attacks. In the case of *S. mutans*, the extracellular matrix is mostly composed of polysaccharides. Nguyen et al. have shown that the amount of produced insoluble exopolysaccharides decreased after treatment with α-MG [49]. This can lead to weakening of the biofilm and its adhesion to the surface. The GtfB and GtfC enzymes, responsible for insoluble glucans, were affected, as well as the amount of intracellular iodophilic polysaccharides that act as storage components in the biofilm matrix. Furthermore, Sivaranjani et al. [22] found that α-MG downregulated key biofilm-associated genes (*bfmR, pgaA, pgaC, csuA/B, ompA, bap, katE*, and *sodB*), including those responsible for extracellular matrix production. α-MG also reduced extracellular polymeric substance secretion, further impairing the structure and protective function of the matrix.

In a 2017 study by Nguyen et al. [51], the effects of α-MG on *S. aureus* biofilm were studied. The MIC for planktonic *S. aureus* strains was 4.58 μmol/L (NCTC 6571 strain), 4.58 μmol/L (MSSA 15981 strain), and 9.15 μmol/L (MRSA 252 strain). The highest MIC was obtained for the MRSA strain, reflecting its resistance profile. To test the effect of α-MG on biofilm, the compound was added at the beginning of biofilm development. For the MRSA strain, the biofilm did not show strong signs of inhibition even after adding 96 μmol/L of α-MG, which is around 10 ×  MIC. However, for the other two strains, smaller amounts of α-MG resulted in the growth inhibition of biofilm. The concentration of 48 μmol/L, which is around 10 × MIC, caused inhibition of biomass at >81.0% for the NCTC 6571 strain and at >93.5% for the MSSA 15981 strain. The observed differences in results for the MRSA strain is the difference in the extracellular polymeric matrix composition. α-MG may interact with proteins in the extracellular matrix of MRSA 252 through hydrophobic and hydrogen-bond interactions, while the polysaccharides in the NCTC 6175 and MSSA 15981 matrices may not have such capability. Thus, the proteinaceous extracellular matrix of MRSA 252 may act as a protective barrier by capturing α-MG and reducing its availability. Furthermore, when tested on a mature (24 h and 48 h old) biofilm of the same strains, no inhibitory activity was found. This proves that the anti-biofilm properties of α-MG are dependent on biofilm age and are strongest in the earliest stages of biofilm formation [51]. Similarly, in the study by Sivaranjani et al. [22], α-MG did not significantly affect established biofilms, reinforcing its role as a preventive rather than disruptive agent.

Overall, α-MG exerts its anti-biofilm effects through multiple complementary mechanisms. It inhibits the initial adhesion of bacterial cells to surfaces, preventing the early stages of biofilm development. Additionally, it interferes with the synthesis of extracellular polymeric substances, which are essential for biofilm structural integrity. At the molecular level, α-MG downregulates the expression of key biofilm-associated genes, which encode components involved in matrix production and cell–surface adhesion. Moreover, the compound disrupts bacterial membrane integrity, compromising cellular viability within the biofilm and increasing the accumulation of antimicrobial agents. These combined actions contribute to both the prevention of new biofilm formation and the weakening of pre-existing biofilm structures.

In conclusion, the influence of α-MG on single-species biofilm depends on the age and the developmental stage of the biofilm. Its anti-biofilm activity is primarily observed in the early stages of biofilm formation and is associated with interference in the adhesion of bacteria to the surface.

#### 2.3.2. Multi-Species Biofilms

Previously mentioned studies focused on single-species biofilms; however, these types of biofilms are rarely found in nature. Studies on multi-species bacterial biofilms give more insight into the actual possibilities of anti-biofilm applications. Nguyen et al. [52] investigated a biofilm comprising three bacterial strains, simulating dental plaque: *Streptococcus mutans*, *Streptococcus oralis*, and *Actinomyces naeslundii*. The α-MG treatment involved four brief, one-minute exposures, which led to a 30% reduction in overall biofilm biomass and a notable shift in its microbial composition. The relative abundance of *S. mutans* decreased from 72% to 57%, *A. naeslundii* was almost completely eradicated, while *S. oralis* exhibited an increase in proportion. α-mangostin was found to inhibit glucosyltransferase enzymes involved in extracellular polysaccharide synthesis. Furthermore, confocal microscopy images confirmed α-MG accumulation within the biofilm matrix, which suggests the potential for sustained antimicrobial activity after a brief exposure.

In a 2022 study, Leelapornpisid [53] proved the anti-biofilm action of α-MG on a multi-species bacterial–fungal biofilm model. In this study, a biofilm consisting of three bacterial strains (*Enterococcus faecalis*, *Lactobacillus rhamnosus*, and *Streptococcus gordonii*) and one yeast strain (*Candida albicans*) was developed and allowed to mature for 48 h. This multi-species biofilm was designed to mimic the conditions of endodontic infections. Exposure to 0.2% α-MG for 24 h significantly reduced the metabolic activity and viability of the biofilm. Compared to 2% chlorhexidine, α-MG showed similar biofilm-disrupting activity but additionally suppressed hyphal formation in *C. albicans*, altering fungal morphology. α-MG also interfered with cell membrane integrity and inhibited key glycolytic enzymes such as aldolase, glyceraldehyde-3-phosphate dehydrogenase, and lactic dehydrogenase.

α-MG is an effective compound in combating multi-species biofilm. In the case of bacterial–fungal biofilms, it causes inhibition of the glucosyltransferase enzyme involved in the extracellular synthesis of polysaccharides, and in fungi, it also suppresses hyphal formation.

### 2.4. Drug Delivery Strategies

Research into novel antibacterial and anti-biofilm agents must also address the challenge of efficient drug delivery. An important challenge for α-MG’s use in pharmaceuticals is its low solubility in water of around 2 μg/mL [54], which causes low bioavailability [55]. This prompts researchers to seek alternatives that would allow for the use of α-MG in pharmaceuticals without relying on high concentrations of alcohol or surfactants, which are used as solubilizers but increase toxicity [56].

One way of improving bioavailability is synthesizing an emulsion containing α-MG particles. Such a nanoemulsion was prepared by Asasutjarit et al. using ultrasonication and oil base consisting of several mixed oils (light mineral oil, cyclomethicone, dimethicone, jojoba oil, and caprylic/capric triglycerides) [56]. The nanoemulsion exhibited steady release of α-MG over time after initial quick release in the first 30 min of testing. Furthermore, the nanoemulsion containing α-MG succeeded in inhibiting *S. aureus* and *P. acnes* growth and presented very low toxicity against skin cells. In another study of α-MG nanoemulsion, an application as an irrigant for treating infected root canals was tested [57]. Palm-oil-based nanoemulsion with a 0.2% concentration of α presented strong antibacterial and antifungal action against tested *E. faecalis, S. epidermidis*, and *C. albicans* biofilms. Other researchers also succeeded in creating nanoemulsions with α-MG [58]. These studies combined suggest that nanoemulsions can be an effective way of increasing α-MG’s bioavailability, and such products could even replace commonly used substances such as chlorhexidine and NaOCl [57].

For better physical stability, emugels, which are emulsions with the addition of thickening agents, such as polymers to form a gel-like substance, can be used [59]. Some studies also suggest a more controlled release, bioavailability, and skin absorption rates for emugels compared to emulsions [60]. In a study by Sungpud et al., a nanoemulsion loaded with mangostin extract, containing α-MG, β- and γ-mangostin, was tested against emugel and nanoemugel based on this emulsion and thickened with oligosaccharide. Tested nanoemulsions exhibited antibacterial action against *S. aureus* and *E. coli* with MIC values of mangostin extract ranging from 790 to 1560 μg/mL, which was noticeably lower than MIC for bulk mangostin extract with the same extractants used (1 560–3130 μg/mL). Nanoemugel had the best drug release characteristics, with a drug release of 87–94% compared to 74–78% for emugel. Combined with higher viscosity and improved skin retention compared to nanoemulsions, emugels and nanoemugels can be used for topical applications in cosmetic formulations.

Liposomes, self-assembling multilayer lipid-based drug carriers with an aqueous center, have been used for drug delivery because of their biocompatibility and protection of the enclosed drug. They are especially useful for hydrophobic drugs like α-MG because the encapsulated drug has improved aqueous solubility [61]. They can also be used in targeted drug delivery, minimizing side effects and increasing the maximum non-toxic dosage [62]. Liposome carriers have been used for α-MG delivery for the hepatocellular carcinoma Hep-G2 cell line in a study by Phan et al. [63]. The liposomes were synthesized using the thin-film hydration method, followed by extrusion through a polycarbonate membrane to achieve a uniform size of the particles. Encapsulated α-MG maintained a steady release over 96 h, showing the stability and potential for long-lasting treatment with such solution. Another liposome-based application was presented by Benjakul et al., who tested α-MG loaded liposomes, prepared with the reverse-phase evaporation method, against four lines of human carcinoma cells [64]. The liposome bilayers were formulated from a lipid mixture of phosphatidylcholine and cholesterol. The use of liposome carriers significantly improved α-MG’s aqueous solubility and achieved drug entrapment of 81%, while enabling controlled drug release. This proved the liposomes to be a useful solution for hydrophobic drug delivery.

Previously mentioned studies show a general use of liposome carriers for α-MG. The antibacterial properties of such particles were also tested by Kim et al. in a 2024 study [65]. Nanoliposomes were made from ecithin-based vesicles with the addition of casein and loaded with mangosteen extract and γ-cyclodextrin. The particles were tested against various *Staphylococcus* species, with the lowest MIC values of liposomes exhibited for *S. pseudintermedius, S. felis*, and *S. schleiferi* (55 μg/mL of liposomes tested by broth microdilution, approximately 1 μg/mL of α-MG). When tested against clinical isolates from skin diseases of companion dogs and cats, MIC values of the loaded liposomes for 50% of *Staphylococcus* isolates ranged from 110 μg/mL (approximately 2 μg/mL α-MG) for *S. pseudintermedius* to 880 μg/mL (16 μg/mL of α-MG) for *S. aureus*. These results confirm liposome viability for antibacterial and wound healing applications.

Another method of creating drug carriers involving lipids is synthesizing nanostructured lipid nanoparticles, which are made with biodegradable physiological lipids, surfactants, and co-surfactants [66]. Such carriers have high physical stability and allow for controlled drug release. Some studies show the potential of creating nanostructured lipid carriers loaded with α-MG in combination with other phytochemicals. One such application was the synthesis of nanoparticles containing α-MG and propolis [67]. Both propolis and α-MG are hydrophobic, so this solution makes them both more biocompatible and allows for increased aqueous solubility (13 times compared to free α-MG). In another study, the lipid nanocarriers contained α-MG and clove oil in a liquid lipid phase inside the core of the nanoparticle [68]. This method resulted in increased inhibition of bacterial growth for Gram-negative bacteria, including *E. coli* and *K. pneumoniae*, compared to antibiotics, free α-MG, and clove oil as well as the clove oil and α-MG mixture. The same can be said for Gram-positive bacteria, including three strains of *S. aureus*. The created nanoparticles were stable, sustaining their properties for up to 60 days. The drug release was steady over 72 h, and the drug penetration was confirmed on an ex vivo canine gingival model with penetration of 100 μm within 45 min when using a spray containing the nanoparticles. The findings of this study indicate that the proposed approach holds promise as an alternative therapeutic strategy against a wide spectrum of both Gram-positive and Gram-negative bacteria.

Other materials, besides lipids, can be used for creating α-MG loaded nanoparticles. In a 2021 study, Nguyen et al. [69] investigated the possibility of synthesizing polymeric, α-MG loaded nanoparticles for anti-biofilm treatment. The efficiency of the nanoparticles was tested against *S. aureus* MSSA and MRSA strains. The study demonstrated that α-MG-loaded nanoparticles were significantly more effective than free α-MG in inhibiting biofilm formation, achieving biomass reductions of 62% against the reference *S. aureus* NCTC657 strain and 53% against MRSA252 at a concentration of 24 μmol/L. At 48 μmol/L, the nanoparticles nearly eradicated the early-stage biofilms. While their effect on mature 24 h biofilms was limited with a 22–27% biomass reduction, they significantly inhibited bacterial adhesion and killed embedded cells. Gene expression analysis confirmed the downregulation of key biofilm-associated genes with strain-dependent variations. These findings highlight α-MG-loaded nanoparticles as a promising strategy for biofilm prevention, particularly in the early stages of biofilm formation.

Cellulose-based α-MG-loaded nanoparticles were tested against acne vulgaris [70]. The planned therapy was based on follicular penetration in the sebaceous gland area, and the size of the nanoparticles was suited for this application. Antibacterial activity was confirmed for *P. acnes* with the α-MG MIC of 15.625 μg/mL, which was lower than for free α-MG in a water solution (>250 μg/mL).

Another proposed solution for localized drug delivery targeting biofilms is soluble hydroxypropyl methylcellulose-based α-MG films developed by Tangsuksan et al. [34]. This soluble film was evaluated against key oral pathogens linked to biofilm-mediated diseases, including *S. mutans*, *Porphyromonas gingivalis*, and *Candida albicans*. Treatment using this approach has shown a time-dependent bactericidal and fungicidal effect, achieving complete microbial elimination within 24 h. While the anti-biofilm action was moderate, the film’s capacity to adhere to oral tissues and sustain the continuous release of α-MG over the course of treatment indicates promising potential for clinical applications in the prevention of dental plaque and candidiasis. Another study proposed using a hydrogel mucoadhesive film loaded with α-MG and γ-cyclodextrin as a solution for α-MG’s delivery [71]. The results of applying the film to oral mucosa showed increased mucoadhesive force and time, as well as increased retention time and improved wound healing efficacy in a rat model of recurrent aphthous stomatitis, compared to films containing α-MG alone or a physical mixture of α-MG and γ-cyclodextrin. A similar mucoadhesive film was tested for aphthous stomatitis in another study [72] with good results. This further supports mucoadhesive films combination with α-MG as a promising treatment option for oral mucosal disorders.

Shahzad et al. obtained a similar effect by testing hydrogel membranes loaded with α-MG [73]. The membranes were comprised of gellan gum, glycerol, and a surfactant with different concentrations of α-MG to find the optimal formulation. The hydrogel membrane enhanced its aqueous solubility, prolonged the release profile, significantly improved its antibacterial, antioxidant, and anti-inflammatory efficacy while reducing cytotoxicity and enabling better tissue regeneration in in vivo tests, compared to free α-MG and blank membranes.

A combined therapy strategy has been suggested by Wojnicz et al. in a study related to α-MG’s influence on photodynamic therapy outcomes [74]. α-MG has been shown to enhance membrane permeability and inhibit efflux pump in bacteria, thereby facilitating photosensitizers’ accumulation in cells. The accumulation was confirmed through digital holotomography and confocal microscopy, which revealed increased refractive index values and fluorescence intensity. This suggests that α-MG has potential as an enhancer in photodynamic therapies, potentially improving the efficacy of conventional treatment methods.

A major challenge for the pharmaceutical industry is the low aqueous solubility of α-MG. Current studies using nanoemulsions and encapsulation of α-MG in liposomes appear to be effective solutions for this problem.

### 2.5. Synergistic Effects

Despite its broad-spectrum antibacterial effects, α-MG’s combination with conventional antibiotics or other phytochemicals has been shown to significantly enhance its efficacy, particularly against very resilient bacteria. Synergistic solutions, combining α-MG with other bactericides, are studied for an even stronger effect. Such synergistic strategies can overcome bacterial defense mechanisms and restore antibiotic sensitivity. Several studies show good results after combining α-MG with already known antibiotics that the bacteria might have become resistant against. Some mechanisms of this synergistic action will be described more closely in this section.

Plant-derived compounds can synergistically interact with other components of extracts, as well as other antibacterial substances such as antibiotics.

#### 2.5.1. Overview of Synergistic Mechanisms

The synergy between α-MG and other agents arises from multiple, complementary mechanisms:

- Increased membrane permeability: α-MG disrupts bacterial membrane integrity, which facilitates the intracellular uptake of co-administered antibiotics. This has been further described above as shown by Koh et al. [15] and other groups. Since antibiotic resistance can be based on bacteria’s limited uptake of a drug, this mechanism can help overcome the boundary and allow for antibiotics’ proper action [75].
- Efflux pump inhibition: Increased multi-drug efflux pump is one of the most common mechanisms of induced bacterial resistance [75,76]. Studies have shown that α-MG can downregulate the expression of efflux pump-related genes or interfere with their function, reducing antibiotic clearance from the cell [17].
- Enzyme inhibition: Another mechanism of bacterial antibiotic resistance is ability to destroy the antibiotic molecule. This is the main mechanism for β-lactam resistance [76]. In resistant strains, such as MRSA and oxacillin-resistant *Staphylococcus saprophyticus* (ORSS), α-MG has been shown to inhibit resistance-related enzymes like β-lactamase and PBP2a, restoring the efficacy of β-lactam antibiotics [14,17].
  Together, these mechanisms cam explain the observed synergistic effects of α-MG and antibiotics in both planktonic and biofilm-forming bacterial populations.

#### 2.5.2. Synergy with Other Bactericidal Drugs

The synergistic antibacterial potential of α-MG has been demonstrated in multiple studies involving common antibiotic-resistant bacterial strains. The studies show that the compound enhances the efficacy of common antibiotics and allows for treatment in cases where the bacterial resistance could prevent it.

One of the earliest demonstrations of this potential was presented by Sakagami et al. [32], who demonstrated an improved antibacterial activity against vancomycin-resistant *Enterococci* (VRE) and MRSA after combining α-MG with commercially available antibiotics (ampicillin, gentamicin, minocycline, and vancomycin hydrochloride). These findings suggested that α-MG may help restore antibiotics bactericidal action for resistant strains that pose a common threat in the clinical environment.

In a related study, Phitaktim et al. focused on the effects of α-MG against oxacillin-resistant *Staphylococcus saprophyticus* (ORSS) [14]. *S. saprophyticus* has reportedly become resistant to many β-lactam antibiotics, posing a significant challenge in treating urinary infections. The study demonstrated that α-MG exhibited moderate antibacterial activity with a MIC of 8 μg/ml, while oxacillin alone was significantly less effective (MIC = 128 μg/mL). However, the combination of α-MG with the antibiotic has improved activity against ORSS, confirming a synergistic effect. Further mechanistic studies revealed that α-MG inhibited β-lactamase type IV from *Enterobacter cloacae* in a dose-dependent manner, suggesting that it may help restore oxacillin efficacy by reducing enzymatic degradation. TEM and confocal laser scanning microscopy images showed that the combination of α-mangostin and oxacillin caused abnormal cell morphology and leakage of intracellular DNA as a result of cytoplasmic membrane disruption and increased membrane permeability.

Building on the synergistic results of previously conducted studies, Ge et al. [17] went through a more structural route in their 2024 study. In this work, a derivative of α-MG, termed α-MG-4, was synthesized to enhance α-MG’s antibacterial properties and reduce cytotoxicity. This compound, characterized by the addition of an allyl group at the C-3 position of the xanthone scaffold, exhibited markedly stronger synergistic effects when paired with antibiotics like penicillin, enrofloxacin, and gentamicin compared to the parent compound. The synergistic effect was validated in vivo using a mouse skin abscess model with the combination of α-MG and penicillin resulted in a significant reduction in abscess size and inflammation.

The underlying mechanisms of this synergistic action were further elucidated through advanced imaging techniques such as SEM and TEM. These analyses revealed that α-MG-4 has increased the bacterial membrane permeability and inhibits the expression of efflux pump-coding genes. Such modifications lead to increased intracellular accumulation of antibiotics, amplifying the bactericidal action. Moreover, α-MG-4 inhibited the expression and activity of PBP2a, a key factor in MRSA β-lactam resistance [17].

Shifting from clinically critical pathogens typically associated with hospital-acquired or invasive infections, Ahmad et al. [33] investigated the synergistic potential of α-MG against skin-associated bacteria commonly implicated in mild but persistent conditions such as acne. The study examined three antibiotics—tetracycline, erythromycin, and clindamycin—that are widely used to treat skin diseases. However, in recent years, their effectiveness has decreased due to the growing resistance of various bacterial strains. The combination of antibiotics with α-MG demonstrated effectiveness against *Propionibacterium acnes*, *S. aureus*, *S. epidermidis*, and *S. pyogenes*. Inhibition of bacterial growth was observed within 10–12 h after applying the combined treatment. In contrast, treatment with α -MG or antibiotics alone did not result in complete inhibition, even after 24 h of exposure. These findings show the enhancement and acceleration of the antibacterial action when combined therapy is used.

Some studies have shown synergistic activity between α-MG and other phytochemicals [19,77]. In a 2019 study by Samprasit et al. [77], synergy between α-MG and resveratrol was investigated. Resveratrol is a natural compound produced by grapes and mulberries with moderate antimicrobial activity. In the study, both phytochemicals have been combined into polymer-based wound dressing films and tested in vitro. The combination of compounds exhibited enhanced bacterial inhibition even at sub-MIC concentrations, which was further confirmed by time–kill assays showing significant reductions in bacterial viability. The underlying mechanism of synergy may involve complementary modes of action and improved permeability. These findings highlight the therapeutic potential of combining α-MG and resveratrol in a biodegradable film matrix for the treatment of infected wounds, particularly those involving antibiotic-resistant bacterial strains.

Another phytochemical with synergic action with α-MG is lawsone methyl ether, derived from *Impatiens balsamina* as shown by Meah et al. [19]. When used in combination with α-MG-rich extract, it produced potent synergistic effects against MRSA. The combination significantly increased bacterial membrane disruption and permeability, facilitating the intracellular accumulation of antibacterial agents.

Collectively, these studies highlight the potential of α-MG as a promising agent in combination therapies against antibiotic-resistant bacterial strains, paving the way for effective treatment strategies for bacterial infections.

α-MG can be combined with commonly used antibiotics to restore the effectiveness of treatment against drug-resistant strains. The synergistic effect varies depending on whether Gram-positive or Gram-negative bacteria are targeted, with combinations of α-MG and antibiotics showing greater efficacy against Gram-positive bacteria.

## 3. Conclusions

Growing antimicrobial resistance is a risk to the general health of people, animals, and the environment since microorganisms, such as bacteria, spread to all economic sectors. Therefore, the solution to the problem should be found through integrated multi-sector action known as One Health. One of the strategies is searching for new substances of natural origin that could be used as antibacterial drugs active against resistant bacteria. Our work is focused on the review of the available literature containing information about the applications of α-MG as a natural agent in combating multidrug-resistant bacteria. The presented data are promising for the researchers and clinicians aiming to develop innovative methods of counteracting resistant infections. The provided information can facilitate the transition from in vivo to in vitro studies. Our study is a valuable foundation for future clinical trials involving α-MG.

A deeper understanding of the mechanisms of action of α-MG, a plant-derived compound, presents the opportunity for its chemical modification to enhance the effectiveness of therapy against resistant bacterial strains. Particular attention should be given to the use of α-MG in combination with conventional therapeutics that have traditionally been used for treating infections. In the future, α-MG could serve as a co-therapeutic and a dose-modifying agent by interacting with altered cellular targets or inhibiting bacterial resistance mechanisms. This would enable the reuse of known antibiotics against bacteria that have developed resistance, especially in the treatment of multidrug-resistant Gram-positive bacteria.

Various approaches have been developed to reduce infections and biofilm development. α-MG exhibits excellent antibacterial properties at relatively low concentrations, particularly against Gram-positive bacteria. Its mechanisms of action include membrane disruption, inhibition of essential metabolic pathways, and downregulation of biofilm-related genes. It interferes with bacterial adhesion, disrupts existing biofilm, and its extracellular matrix. However, α-MG’s limited activity against Gram-negative bacteria remains a major challenge for applications in antibacterial therapy.

Other challenges include α-MG’s poor solubility, rapid elimination, and poor pharmacokinetic properties, which have hampered its use as a drug, and it remains only a supplement in the pharmaceutical market. To address this problem, many techniques have been used, including the development of nano-delivery systems. α-MG’s nanoformulation improves the dispersion of α-MG in aqueous solutions. Benefits of this approach include higher bioavailability, prolonged stable drug release, and increased stability. Conjugation of α-MG nanoparticles with targeting ligands such as peptides and aptamers could provide specific targeting; however, this method is currently mostly used in cancer treatment studies.

Moreover, synergistic combinations with antibiotics and other phytochemicals offer promising strategies to combat multidrug-resistant infections. Combining antibiotic treatment with α-MG’s membrane-disrupting and efflux pump-inhibiting properties enhances antibacterial efficacy, allowing for smaller drug doses and restoring antibiotic susceptibility in resistant strains. The synergistic antibacterial effects of α-MG and resveratrol have been demonstrated, and drug delivery systems combining α-MG with clove oil or propolis have yielded promising results. Through various research directions, scientists can improve the bioavailability and therapeutic properties of α-MG to achieve high therapeutic efficacy.

Other antibacterial mechanisms, not yet proven for α-MG, should be further researched. This includes the generation of reactive oxygen species (ROS), which can kill the bacterial cells by degrading intracellular DNA and lead to cell apoptosis [78,79]. α-MG’s ability to generate ROS has been proven in the context of cancer treatment and is widely described in the literature [80,81]. However, the antibacterial applications of this effect of α-MG have not been studied.

Another mechanism that could be studied in the future is quorum sensing inhibition. This mechanism interferes with the coordination of bacteria while forming biofilm and influences the virulence and pathogenesis of bacteria. Quorum sensing can be disrupted by regulating related genes [78]. One study suggested that α-MG can inhibit quorum sensing in bacteria, based on results against *Chromobacterium violaceum* [82]. However, no further studies were found in relation to this mechanism. Future research could prove insightful.

Despite these promising results, further research is needed to standardize extraction methods, optimize drug delivery platforms, and fully characterize the pharmacokinetics, toxicity, and clinical efficacy of α-MG. Continued efforts in these areas are essential to facilitate the translation of α-MG into effective antimicrobial therapies.

## Figures and Tables

**Table 1:** Search results for queries involving α-mangostin and antibacterial activity from 2020 to 2025. Results were obtained with the use of Publish or Perish (Harzing, 2024) [8].

Query	Source	Papers	Citations	Cites per Year
alpha-mangostin [title] antibacterial; bacteria	Google Scholar	51	422	84.40
alpha-mangostin [title] antibacterial; biofilm	Google Scholar	17	177	35.40
alpha-mangostin; antibacterial	PubMed	29	0	0.00
alpha-mangostin [title] antibacterial	PubMed	11	0	0.00
alpha-mangostin [title] antibacterial; biofilm	PubMed	1	0	0.00
